# A Driving-Adapt Strategy for the Electric Vehicle with Magneto-Rheological Fluid Transmission Considering the Powertrain Characteristics

**DOI:** 10.3390/s22249619

**Published:** 2022-12-08

**Authors:** Peng Liao, Donghong Ning, Tao Wang, Haiping Du

**Affiliations:** 1 College of Engineering, Ocean University of China, Qingdao 266110, China; 2 School of Vehicle and Mobility, Tsinghua University, Beijing 100084, China; 3 School of Electrical, Computer and Telecommunications Engineering, University of Wollongong, Wollongong, NSW 2522, Australia

**Keywords:** electric vehicle, magneto-rheological fluid transmission, driving-adapt, driving strategy, powertrain

## Abstract

The additional energy consumption caused by the incompatibility between existing electric vehicle (EV) powertrain characteristics and driving conditions inevitably curbs the promotion and development of EVs. Hence, there is an urgent demand for the driving-adapt strategy, which aims to minimize EV energy consumption due to both powertrain characteristics and driving conditions. In order to fully explore the EV driving-adapt potential, this paper equips the EV with a magneto-rheological fluid transmission (MRFT). First, an EV dynamics analysis of the driving conditions, the powertrain model considering the energy transmission process, and the driving-adapt transmission model considering magneto-rheological fluid (MRF) is conducted to clarify the quantitative relation between the driving conditions and the powertrain. Second, a driving-adapt optimization strategy in the specific driving condition is proposed. Finally, the results and discussions are executed to study (i) the determination of the MRFT fixed speed ratio and variable speed ratio range, (ii) the application potential analysis of the proposed strategy, and (iii) the feasibility analysis of the proposed strategy. The results indicate that (i) the urban driving condition has higher requirements for the MRFT, (ii) EVs equipped with MRFT achieve the expected driving performance at the most states of charge (SOCs) and environmental temperatures, except for the SOC lower than 10%, and (iii) the driving time with efficiency greater than 80% can be increased by the MRFT from 10.1% to 58.7% and from 66.8% to 88.8% in the urban and suburban driving conditions, respectively. Thus, the proposed driving-adapt strategy for the EV equipped with the MRFT has the potential to alleviate or eliminate the traffic problems caused by the incompatibility of the EV powertrain characteristics and the driving conditions.

## 1. Introduction

Vehicle electrification, an inevitable method to realize green transportation, has revealed a great potential to solve traffic problems, such as environmental pollution, energy consumption, and so on [1,2]. However, the increasingly acute traffic problems derived from the existing operating characteristics of the electric vehicle (EV) powertrain in a specific driving area have seriously hampered the promotion and development of EVs [3]. These traffic problems, including excessive EV energy consumption, limited driving mileage, EV driving performance degradation, and so on, are particularly evident in specific driving conditions. In addition, the performance degrees of these problems show significant differentiation once the driving conditions change [4].

The EV powertrain is usually composed of the battery, the electromotor, the transmission, and other transmission devices [5]. For the sake of describing the battery energy consumption, researchers mostly adopt the equivalent circuit model (Rint model, RC model, PNGV model, and so on) to model and analyze the commonly used batteries (lead-acid battery, nickel-hydrogen battery, and lithium-ion battery) of EVs [6,7]. The internal resistance of the battery is divided into ohmic internal resistance, electrochemical internal resistance, and concentration polarization internal resistance [8]. Relevant research shows that, based on the battery principle, the battery equivalent circuit model, which is composed of resistors, can be applied to model the energy consumption of many kinds of batteries [9]. Furthermore, in order to describe the highly nonlinear characteristics of battery performance, scholars used a neural network battery model to explore the operation performance of lithium batteries [10]. In addition, the electromotor equipped for EVs and the recovery of braking energy are also the focus of researchers [11]. Based on the power transmission process and considering the operation characteristics of the electromotor, scholars constructed the evaluation parameters of braking energy recovery, which verified the feasibility and effectiveness of the evaluation method [12]. At the same time, some scholars have adopted the method of adjusting the transmission speed ratio to improve the recovery efficiency of the braking energy of EVs [13]. However, most of the above research related to the EV powertrain optimize the powertrain for specific driving performance (power, economy, environmental protection, etc.), while ignoring the external EV motion demands on the powertrain control optimizations.

As for the driving conditions, with the continuous development of urbanization, the daily travel of residents tends to be diversified and specialized [14,15]. This illustrates that EV driving conditions are getting more and more stable, and that most driving time is accounted for by specific driving conditions, such as the urban driving condition or the suburban driving condition [16]. In that case, the driving regions of most EVs are relatively unchanged and have strong regional characteristics. Indeed, realistic driving conditions are always affected by such factors as people, vehicles, roads, and environment, showing complex real-time changes. In order to characterize the driving conditions, researchers have proposed a series of driving conditions, such as the New European Driving Cycle (NEDC), the Worldwide harmonized Light vehicles Test Procedure (WLTP), and so on [17]. Notably, the NEDC includes four urban cycles and one suburban cycle, and the detailed velocity profiles in the urban and suburban driving conditions can be obtained by the corresponding cycles. The urban driving condition lasts for 780 s, and the average speed of the vehicle is 19 km/h. In contrast, suburban driving lasts for 400 s conditions, and the average speed of vehicles is 62.6 km/h. Many studies utilize these driving cycles to explore the driving performance of vehicles under different control strategies, powertrain structures, and so on [18]. However, this research relevant to EVs is always focused on specific performance, such as the maximum driving velocity, the shortest acceleration time from 0 km/h to 100 km/h, etc., and ignores EV performance under specific driving conditions. Moreover, once the EV powertrain is built, its characteristic adjustment is too expensive to adapt to the drivers’ personalized driving conditions.

Fortunately, with the rapid development of intelligent materials and machinery manufacturing, magneto-rheological fluid transmission (MRFT) technology has shown great potential to realize efficient, high-speed, accurate, and adjustable transmission processes, and has contributed intelligent power to realize the adaptation of EV powertrain characteristics to specific driving conditions [18]. The MRF has the ability to change its yield strength in the millisecond range under the action of the magnetic field. Under the action of no external magnetic field, the MRF behaves as a Newtonian fluid. In contrast, the MRF changes from free flow without a magnetic field to solid-like flow, showing the characteristics of Bingham fluid with high viscosity, under the action of the external magnetic field [19,20]. Therefore, MRFT technology has the advantages of both easy and stepless speed ratio regulation, simple control, fast response, and small volume [21]. In this context, the introduction of MRFT technology into the EV powertrain can make full use of the solid-liquid two-phase nature of the MRF to achieve flexible transmission of EV power and meet the differential driving needs. Taking MRF as the transmission medium in the transmission can not only continuously adjust the state of power, but also effectively realize the efficient and flexible transmission of torque and speed, which has far-reaching development significance and application prospects.

Fully considering the existing studies, even these studies obtain many thought-provoking results focusing on EV driving scenarios or EV powertrain characteristics, but few of them pay attention to the increasingly stable driving conditions and the adjustability of MRFT technology [22,23]. In this context, making full use of the transmission regulating effect on the EV powertrain reveals the quick and accurate adaption to specific driving conditions. Thus, deeply exploring the optimization potential brought by MRFT technology in specific driving conditions can comprehensively improve the driving performance of EVs from both design and application aspects, and alleviate or solve existing problems caused by EV powertrain characteristics.

The remainder of this paper is organized as follows: Section 2 analyzes EV dynamics in different driving conditions, constructs the powertrain model considering the energy transmission process, and then proposes the driving-adapt transmission model considering MRF. Section 3 describes the driving-adapt optimization strategy in specific driving conditions. Then, some results and discussion are conducted in Section 4, which includes the determination of the MRFT fixed speed ratio and variable speed ratio range, the feasibility analysis of the proposed strategy, and the application potential analysis of the proposed strategy. Finally, the main conclusions and future research directions are presented in Section 5.

## 2. Model and Analysis

### 2.1. EV Dynamics Analysis in Driving Conditions

With the rapid development of urbanization, residents’ choice of transportation mode tends to be diversified and specialized. In addition, the clean electric energy of EVs endows the EVs with significant adaptation to daily trips [24,25]. In that case, making full use of the EV driving conditions can tap the EV’s optimization potential from the application level.

**Figure 1:**
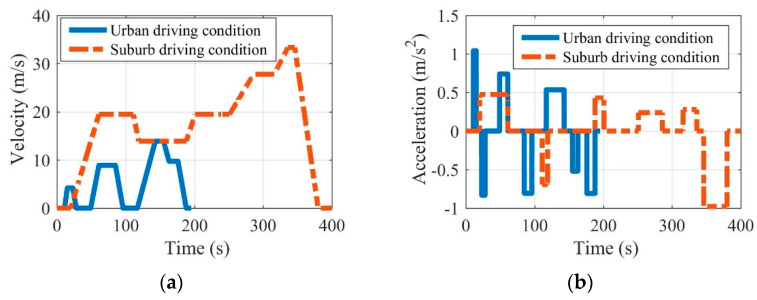
EV velocity (**a**) and acceleration (**b**) under the urban driving condition and the suburban driving condition.

In realistic driving conditions, EV driving states are inevitably affected by factors such as people, vehicles, roads, and the environment. Thus, EV driving states show complex real-time changes and can be obtained by the personalized historical trajectory data of EVs. Taking the EV driving area into consideration, this paper roughly divided the driving conditions into the urban driving condition and the suburban driving condition according to the NEDC, which contains 4 urban driving conditions and 1 suburban driving condition [26,27]. With the consideration of NEDC, the EV velocity and acceleration in both the urban and suburban driving conditions can be defined in Figure 1. Note that the acceleration and velocity of EVs in the driving conditions can be obtained directly by the NEDC. The average velocity in the urban driving condition is 5.14 m/s, and the maximum velocity does not exceed 13.89 m/s, while the average velocity in the suburban driving condition is 17.22 m/s, and the maximum speed does not exceed 33.33 m/s.

The realization of EV motion demands is closely related to EV dynamic characteristics. In the EV driving process, the driving force of an EV needs to overcome all kinds of driving resistance, i.e., rolling resistance, slope resistance, air resistance, acceleration resistance, and braking force [28,29]. The driving force and braking force, as the force exerted actively by the EV, determine the EV driving states. Based on the force analysis, the calculations of the acceleration and the velocity of the EV are shown in Equations (1) and (2), respectively.

(1) $$a\left(t\right)=\frac{F_{\mathrm{dri}}\left(t\right)-Mgf-Mg\theta -C_{A}v{\left(t\right)}^{2}-F_{\mathrm{bar}}\left(t\right)}{M\delta }$$

where a is the EV acceleration, t is the time instant, $F_{\mathrm{dri}}$ is the needed driving force, M is the EV mass, g is the acceleration due to gravity, f is the coefficient of rolling resistance, θ is the road slope, $C_{A}$ is the aerodynamic drag coefficient, v is the EV speed, δ is the mass factor, and $F_{\mathrm{bar}}$ is the braking force, which is controlled by the brake and limited by the adhesion between the ground and the wheels.

(2) $$v\left(t+\Delta t\right)=v\left(t\right)+\int_{t}^{t+\Delta t}a\left(t\right)dt$$

where Δt is the time step.

Combined with the driving conditions and EV dynamic characteristics, the EV power loss in urban and suburban driving conditions can be obtained by Equation (3), i.e.,

(3) $$P_{\mathrm{dri}}\left(t\right)-F_{\mathrm{bar}}\left(t\right)v\left(t\right)=Mgfv\left(t\right)+Mg\theta v\left(t\right)+C_{A}v{\left(t\right)}^{3}+M\delta v\left(t\right)a\left(t\right)$$

where $P_{\mathrm{dri}}$ is the needed driving power.

**Figure 2:**
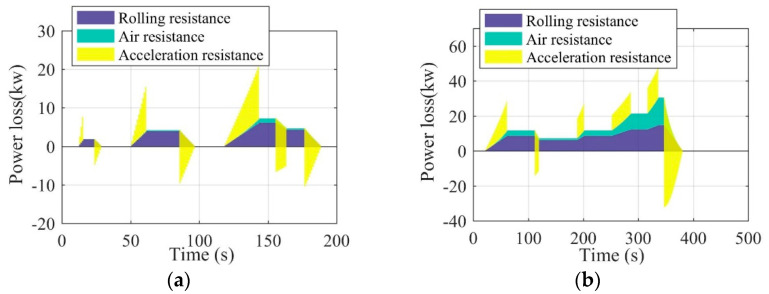
Vehicle power loss under the urban driving condition (**a**) and the suburban driving condition (**b**).

**Figure 3:**
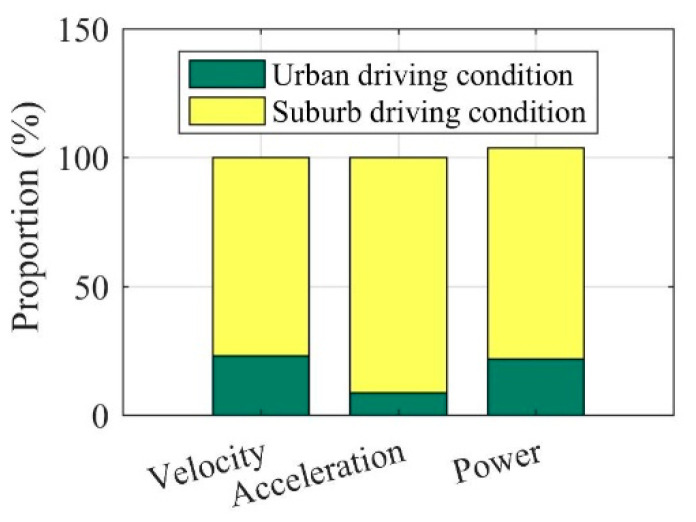
Comparison of EV velocity, acceleration, and power under urban and suburban driving conditions.

Once the acceleration and velocity are determined by the driving conditions, the detailed power analysis can be conducted by the Equations (1)–(3). In the EV driving process, the driving power transmitted from the powertrain to the wheels needs to be balanced with the power loss caused by the driving resistances, as shown in Figure 2. The detailed contrasts can be seen in Figure 3. It can be seen in Figure 3 that there is a huge difference between the urban and suburban driving conditions, where the EV average values of velocity, acceleration, and power under urban driving conditions are far greater than those under suburban driving conditions. These significant differences also confirm the necessity of considering driving conditions in the optimization of the EV driving adaptation.

### 2.2. Powertrain Model Considering the Energy Transmission Process

In order to realize the EV acceleration and velocity in the driving conditions, the powertrain needs to output the rotating mechanical energy with the specific speed and torque through the control of the battery, the electromotor, the transmission, and other power transmission devices.

As the energy supply and storage device of EVs, the operation state of the battery will directly affect the EV driving performance [30,31]. When the power is transmitted in the forward direction, the battery is in the discharge state, and the stored electric energy is transmitted to the electromotor. On the contrary, when the power is transmitted in the backward direction, the battery is charging and will store the electric energy transmitted by the electromotor. Thus, the state of charge (SOC) can be defined as Equation (4), and the battery power can be calculated as Equation (5) according to the battery Rint mode. Note that the SOC is utilized to present the real-time percentage of available electric energy in the battery. Many existing endeavors are executed to maximize the SOC or minimize its change.

(4) $$SOC\left(t+\Delta t\right)=SOC\left(t\right)+\frac{\int_{t}^{t+\Delta t}I\left(t\right)dt}{C_{\mathrm{battery}}}$$

where SOC is the battery state of charge; I is the battery current, where I>0 means the discharging process and others mean the charging process; and $C_{\mathrm{battery}}$ is the original full capacity of the battery.

(5) $$P_{\mathrm{bat}}\left(t\right)=\left\{\begin{array}{l} n_{\mathrm{cel}}\left(U_{\mathrm{oc}}I\left(t\right)-I{\left(t\right)}^{2}r_{\mathrm{dis}}\right),\;\mathrm{if}\;\mathrm{power}\;\mathrm{is}\;\mathrm{in}\;\mathrm{forward}\;\mathrm{direction}\;\\ n_{\mathrm{cel}}\left(U_{\mathrm{oc}}I\left(t\right)-I{\left(t\right)}^{2}r_{\mathrm{cha}}\right),\;\mathrm{if}\;\mathrm{power}\;\mathrm{is}\;\mathrm{in}\;\mathrm{backward}\;\mathrm{direction} \end{array}\right.$$

where $P_{\mathrm{bat}}$ is the battery power, $n_{\mathrm{cel}}$ is the number of battery cells, $U_{\mathrm{oc}}$ is the open circuit voltage of the battery cell, and $r_{\mathrm{dis}}$ $r_{\mathrm{cha}}$ are the discharge and charge resistance of the battery cell, respectively. Note that the adopted values of $U_{\mathrm{oc}}$, $r_{\mathrm{dis}}$, and $r_{\mathrm{cha}}$ can be obtained by the SOC and the battery temperature according to the relevant research [4].

As for the electromotor, it is a power transmission device connecting the battery and the transmission, and its main function is to convert electric energy and rotating mechanical energy through the magnetic field medium [32]. Hence, the real-time convert efficiency can be defined as Equation (6), which is related to its values of torque and speed, i.e.,

(6) $$\eta_{\mathrm{em}}\left(t\right)=f_{\mathrm{em}}\left(n_{\mathrm{em}}\left(t\right),T_{\mathrm{em}}\left(t\right)\right)$$

where $\eta_{\mathrm{em}}$ is the electromotor efficiency; $f_{\mathrm{em}}$ is the electromotor efficiency function, which can be obtained by research [4]; $n_{\mathrm{em}}$ is the electromotor speed; and $T_{\mathrm{em}}$ is the electromotor torque.

With regard to transmission, it is an essential regulating device in the EV powertrain, which provides a new idea for improving EV driving performance through its developments toward automation and the continuously variable speed ratio [33,34]. The speed and torque of the rotating mechanical energy generated by the electromotor in the forward direction or the wheel in the backward direction can be changed by adjusting the speed ratio of the transmission, and the running state of the powertrain can be optimized according to the driving demands. The transmission transmits power through the cooperation of mechanical structures, realizes the specific change of the powertrain transmission ratio, and coordinates the operation status of the whole powertrain. Therefore, the operation characteristics of the transmission are usually expressed as the numerical relationship among transmission efficiency, transmission speed ratio, electromotor torque, and other factors, i.e.,

(7) $$\eta_{t}\left(t\right)=f_{t}\left(i_{t}\left(t\right),T_{\mathrm{em}}\left(t\right),\ldots \right)$$

where $\eta_{t}$ is the transmission efficiency; $f_{t}$ is the transmission efficiency function, which can be obtained in Section 2.3; and $i_{t}$ is the transmission speed ratio.

At present, once the EV powertrain is built, it is difficult for users to adjust it. Hence, with the improvement of driving demands, the widely used powertrain equipped with a stage transmission is hard to adapt to the specific and complex driving conditions of EVs. In addition, the high cost of continuously variable transmission (CVT) is not conducive to the large-scale promotion of EVs. Thus, in view of the close relationship between the transmission speed ratio selection and EV driving performance, this paper takes the transmission as a breakthrough and fully explores the EV powertrain driving adaptability brought by the MRFT technology.

### 2.3. Driving-Adapt Transmission Model Considering MRF

In order to fully explore the EV driving adaptability brought by MRFT technology, this paper qualitatively discusses the operation characteristics of MRFT from a theoretical perspective and compares it with the existing stage transmission and CVT [35,36]. The existing stage transmission usually adopts the form of the multi-speed transmission (MST), which is mainly composed of the gears and has several fixed speed ratios. Due to its simple regulating mode and high efficiency in the fixed speed ratio, it has occupied most of the market share. On the contrary, the CVT can continuously obtain any transmission ratio within the range of the speed ratio, thus the best matching among the powertrain devices can be obtained through CVT adjustment. However, the high cost and relatively low efficiency of the CVT limit its application in the EV powertrain. Fortunately, as an intelligent material, MRF has remarkable rheological properties under the action of the magnetic field, and it provides a broad application space for the powertrain due to its double characteristics of liquid fluidity and solid plasticity.

The application of MRF in the transmission shows that by adjusting the current of the excitation coil, the rheological characteristics of the MRF can be transformed, and then the mutual conversion between the step-less speed ratio and the fixed speed ratio can be realized. Hence, the characteristic of the MRFT is that when the MRF is in the yielding state, the transmission behaves as an MST, while when the MRF is in the unyielding state, the transmission behaves as a CVT. Therefore, the combination of the MST and the MRF can have the advantages of the MST and CVT, that is, high-efficiency transmission in the fixed speed ratio and a specific range of the continuously variable speed ratio. In order to fully explore the application potential of MRFT, this paper constructs an ideal MRFT model for specific driving conditions. The simplest structure of the MRFT can be a joint expansion of MST and MRF. That is, the gear mesh is achieved through MRF rather than gears contact directly.

The speed ratio and efficiency of MST, CVT, and MRFT can be defined as follows, according to the above discussions:

(8) $$\left\{\begin{array}{l} i_{t}\in i_{1},i_{2},i_{3},\ldots ,\;\mathrm{if}\;\mathrm{transmission}\;\mathrm{is}\;\mathrm{MST}\\ i_{t}\in [i_{\min},i_{\max}],\;\mathrm{if}\;\mathrm{transmission}\;\mathrm{is}\;\mathrm{CVT}\;\mathrm{or}\;\mathrm{MRFT} \end{array}\right.$$

where $i_{1}$, $i_{2}$, and $i_{3}$ are the first, second, and third fixed transmission speed ratio; $i_{\min}$ is the minimum transmission speed ratio; and $i_{\max}$ is the minimum transmission speed ratio.

(9) $$\left\{\begin{array}{l} \eta_{\mathrm{MST}}=\eta_{\mathrm{fix}}\\ \eta_{\mathrm{CVT}}\left(t\right)=f_{\mathrm{CVT}}\left(i_{t}\left(t\right),T_{em}\left(t\right)\right)\\ \eta_{\mathrm{MRFT}}\left(t\right)=\left\{\begin{array}{l} \eta_{\mathrm{fix}}-\alpha_{\mathrm{MRFT}}\left(t\right){\left(i_{t}-i_{1}\right)}^{2},\;\mathrm{if}\;i_{t}\left(t\right)\leq i_{1}+i_{1,2}\;\\ \eta_{\mathrm{fix}}-\alpha_{\mathrm{MRFT}}\left(t\right){\left(i_{t}-i_{2}\right)}^{2},\;\mathrm{if}\;i_{1}+i_{1,2}<i_{t}\left(t\right)\leq i_{2}+i_{2,3}\\ \ldots \end{array}\right. \end{array}\right.$$

where $\eta_{\mathrm{MST}}$, $\eta_{\mathrm{CVT}}$, and $\eta_{\mathrm{MRFT}}$ are the efficiency of MST, CVT, and MRFT, respectively; $\eta_{\mathrm{fix}}$ is the efficiency of the gear drive; $\alpha_{\mathrm{MRFT}}$ is the MRFT efficiency coefficient, which is related to the MRF characteristics; $i_{1,2}$ is the efficiency critical point between the first and second fixed transmission speed ratios; and $i_{2,3}$ is the efficiency critical point between the second and third fixed transmission speed ratios

**Figure 4:**
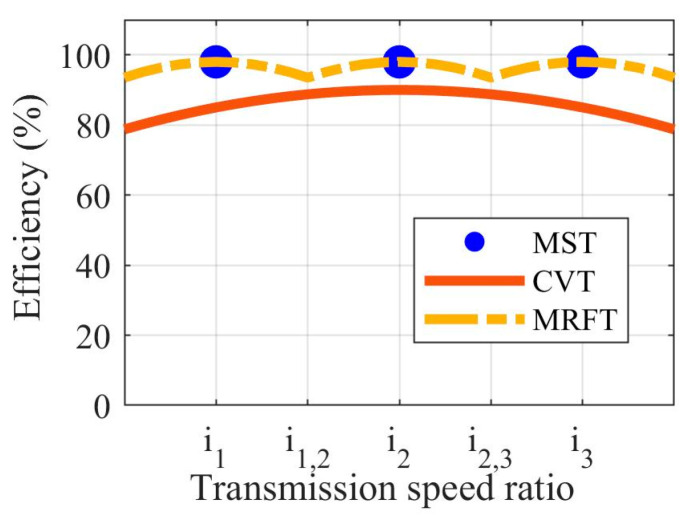
Efficiency diagram of typical transmission, i.e., MST, CVT, and MRFT, under specific operating conditions.

In order to intuitively reflect the driving adaptability mentioned in this paper, the efficiency diagram of typical transmission, i.e., MST, CVT, and MRFT, under specific operating conditions can be seen in Figure 4. The working region of the conceptual MRFT is divided into the fixed speed ratio working region and the stepless speed ratio working region, according to the gear engagement with the help of MRF. Note that the adaptability of MRFT is guaranteed by the selection of the MRFT fixed speed ratio and the MRF. Consequently, the MRFT fixed speed ratio and MRF should be determined by the target driving conditions of the EV. That is, the fixed speed ratio and the range of the continuous speed ratio should be matched to the distribution law of the ideal speed ratio in the target driving conditions.

## 3. Driving-Adapt Strategy in Specific Driving Conditions

**Figure 5:**
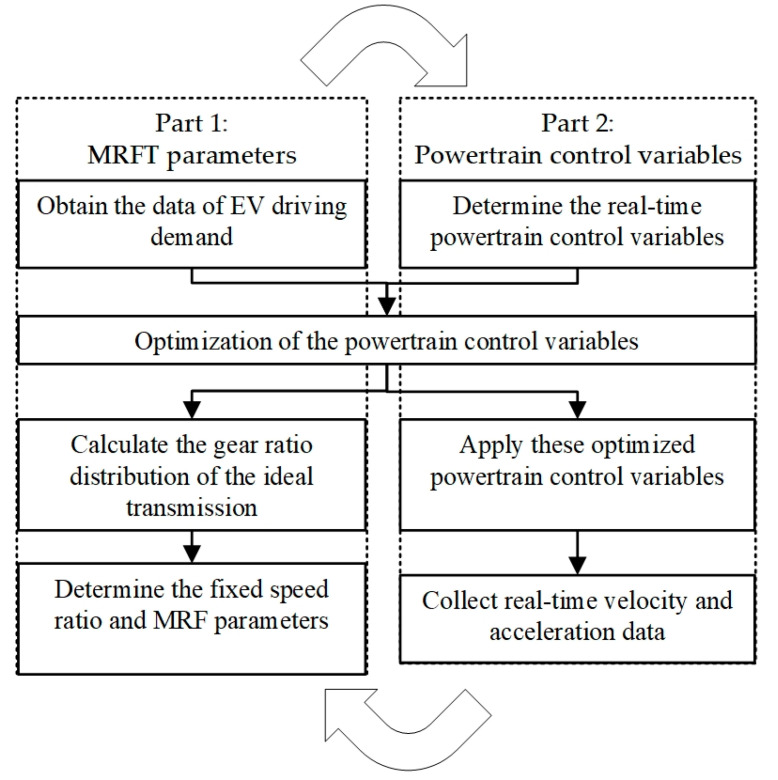
The framework of driving-adapt strategy in specific driving conditions.

The difference in EV driving performance is closely related to the MRFT fixed speed ratio and the powertrain control. In order to achieve the best match between the EV powertrain and the target driving conditions, the MRFT adjustment effect should be utilized to regulate the speed and torque of the rotating mechanical energy. Thus, the MRFT fixed speed ratio should be determined by the target driving condition. Furthermore, the powertrain control is actually the operation state control of the battery, the electromotor, the transmission, and the brake. Therefore, the driving-adapt optimization strategy contains two parts: (1) choose the MRFT fixed speed ratio and the relevant MRF parameters and (2) solve the corresponding control variables of the powertrain in the driving conditions. Note that the fixed speed ratio can be calculated by the optimal powertrain control variables in the corresponding driving conditions, as the MRFT speed ratio is one of the control variables and the fixed speed ratios are the frequent speed ratios with the proper interval. In that case, the proposed strategy is formulated as a global optimization problem, where the output variables are the powertrain control variables once the MRFT fixed speed ratios are decided. The corresponding strategy framework can be seen in Figure 5.

The first part is the determination of the MRFT fixed speed ratio and the relevant MRF parameters. With the rapid development of information technology and the machinery manufacturing industry, MRFT has revealed its great potential in driving adaptation. In the first part, the fixed speed ratio value of MRFT and the relevant MRF parameters can be obtained from the regular changes in the velocity and the acceleration displayed by the driver in the transportation system. This reflects the driving adaptability of MRFT to the drivers’ historical trajectory data. Notably, the periodic implementation of this part can ensure the stable adaptability of EVs to driving conditions by adjusting the fixed speed ratio and the MRF parameters. The detailed steps of the first part are as follows: (1) obtain the data of EV driving demand, that is, obtain the velocity and the acceleration of EVs according to the driver’s driving conditions; (2) calculate the gear ratio distribution of the ideal transmission, that is, calculate the gear ratio of the ideal transmission according to the global optimization strategy; (3) Determine the fixed speed ratio and MRF parameters, that is, choose the fixed speed ratio and the relevant MRF parameters based on the specific requirements.

Once the first part is completed, the EV can formally enter the transportation system to complete the corresponding driving tasks. In this process, the proposed strategy is able to effectively guide or replace drivers to realize the changes in velocity and acceleration through the control of the powertrain. Notably, the real-time trajectory data generated by the EV can be recorded to update the trajectory data or correct the driving conditions in the first part. The detailed steps of the second part are as follows: (1) determine the real-time powertrain control variables according to the driver’s desired velocity and acceleration; (2) Apply these optimized powertrain control variables to achieve the EV desired driving performance; (3) collect real-time velocity and acceleration data in the first part.

Note that both parts of the proposed strategy involve the optimization of the powertrain control variables. Therefore, based on the above analysis, the input variables are the desired real-time EV velocity and acceleration, and the input variables and the output variables can be defined as follows:

(10) $$X\left(t\right)=[v_{\mathrm{des}}\left(t\right),a_{\mathrm{des}}\left(t\right)]$$

where X is the input variables of the driving-adapt strategy, $v_{\mathrm{des}}$ is the desired EV velocity, and $a_{\mathrm{des}}$ is the desired EV acceleration.

(11) $$Y\left(t\right)=[I\left(t\right),i_{t}\left(t\right),I_{\mathrm{MRFT}}\left(t\right),F_{\mathrm{bar}}\left(t\right)]$$

where Y is the output variables of the driving-adapt strategy, and $I_{\mathrm{MRFT}}$ is the MRFT excitation current, which is one of the impact factors of $\alpha_{\mathrm{MRFT}}$.

The proposed optimization strategy should achieve the minimum energy consumption of EVs on the premise of ensuring driving safety. As mentioned before, the SOC is the percentage of available electric energy in the battery. Thus, minimizing the change of it can guarantee minimum energy consumption. Therefore, the objective function of this strategy can be defined as Equation (12):

(12) $$\min\;SOC\left(t\right)-SOC\left(t+\Delta t\right)$$

In order to solve the optimization strategy, the corresponding constraints need to be introduced. For the sake of ensuring the safe operation of EVs, it is also necessary to introduce corresponding safety constraints, i.e.,

(13) $$X_{\min}\leq Xv\left(t\right)\leq X_{\max}$$

where $X_{\min}$ is the minimum values of the input variables, and $X_{\max}$ is the maximum values of the input variables.

(14) $$Y_{\min}\leq Y\left(t\right)\leq Y_{\max}$$

where $Y_{\min}$ is the minimum values of the output variables, and $Y_{\max}$ is the maximum values of the output variables.

At the same time, in order to ensure that the proposed strategy can meet specific driving conditions, that is, to achieve the desired real-time EV velocity and acceleration, it is necessary to introduce corresponding power constraints, i.e.,

(15) Pbattηemtηttηo=Pdrit, if power is in forward direction Pbattηemtηttηo=Pdrit, if power is in backward directionnemt=30ittiovtπR 

where $i_{o}$ is the final reduction drive speed ratio, and R is the wheel radius.

After the determination of the input variables, output variables, objective function, and constraints, the real-time SOC changes can be obtained by determining the driving conditions and different values of output variables based on dynamic programming [37,38]. Combined with the constraints, the optimal control variables can be selected according to the objective function. The detailed steps are as follows: (1) initialization, that is, at the time t, input $X\left(t\right)$ according to the driving requirement and obtain the powertrain working state variables required for calculation; (2) discretization, that is, discretize $X\left(t\right)$ and all possible $Y\left(t\right)$ to calculate all available driving situations with the constraints; (3) choose the optimal output at the ith stage, that is, set the discretized value between $v_{\mathrm{des}}\left(t\right)$ and $v\left(t\right)$ as the corresponding stage, calculate the objective function based on the discrete values, and then choose the optimal values as the output value in the ith stage; (4) update the driving state at the (i + 1)th stage, that is, update the variables related to the objective function calculation; (5) obtain the optimal output variable to achieve the desired driving state, that is, repeat (3) and (4) until the EV driving state satisfies the desired state.

Thus, the proposed DP algorithm can calculate the proposed strategy in the different input variables. As for the algorithm, this paper should give the following note: an error occurs during the discretization, but it will drop when the discrete degrees increase, i.e., the numerical solution is closer to the optimal solution, but the calculation amount rapidly increases.

## 4. Results and Discussions

This section aims to explore (i) the selection of the MRFT fixed speed ratio in specific driving conditions, (ii) the feasibility analysis of the proposed strategy, and (iii) the application potential analysis of the proposed driving-adapt optimization strategy.

The efficiency of MRFT is closely related to the mechanical structure. Therefore, in order to fully explore the driving performance of EVs equipped with MRFT, this paper first determines the fixed speed ratio according to urban and suburban driving conditions. Then, this paper explores the feasibility of MRFT under different SOCs and ambient temperatures. Finally, the driving potential of EVs equipped with MRFT is studied in the contrast to the EVs without transmission. Note that to intuitively display the optimization potential of the MRFT, the power loss of the MRF under different excitation currents is neglected. Hence, this section neglects the determination of the MRF parameters. The relevant simulation parameters are listed in Table 1.

### 4.1. Determination of MRFT Fixed Speed Ratio and Variable Speed Ratio Range

The selection of the MRFT fixed speed ratio and variable speed ratio range should fully consider the EV driving conditions and the influence of the MRFT excitation current. As the MRFT behaves with characteristics of both the MST and the CVT, its efficiency has a high value under the fixed speed ratio, and its speed ratio can fluctuate around the fixed speed ratio by sacrificing part of the efficiency. Therefore, the determination of the fixed speed ratio and the variable speed ratio range can be determined through the following steps: (1) determine the EV driving conditions, (2) calculate the ideal MRFT speed ratio, and (3) determine the fixed speed ratio and the variable speed ratio range according to the distribution of the obtained ideal transmission speed ratio.

**Figure 6:**
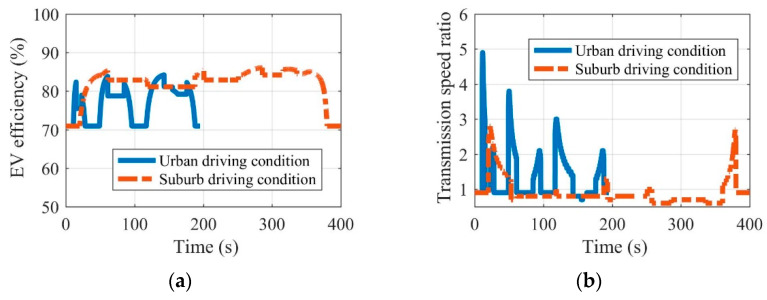
Efficiency and transmission ratio of EV equipped with MRFT under the urban driving condition (**a**) and the suburban driving condition (**b**).

**Figure 7:**
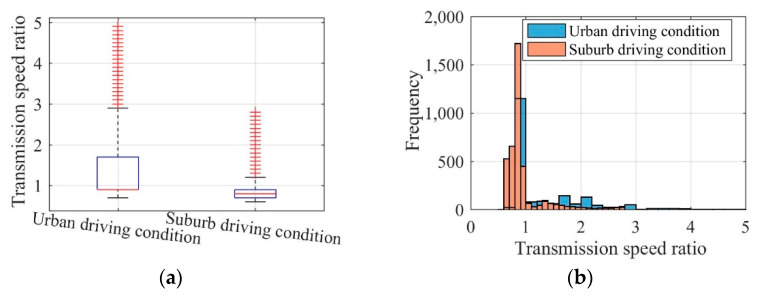
Speed ratio comparison of concept MRFT under the urban driving condition (**a**) and the suburban driving condition (**b**).

Fully considering the difference in driving conditions between urban and suburban areas, this paper takes the urban driving condition and the suburban driving condition in NEDC as an example to explore the determination of the MRFT fixed speed ratio and variable speed ratio range. The efficiency and transmission ratio of EVs equipped with MRFT under the urban driving condition and the suburban driving condition can be seen in Figure 6. The speed ratio comparisons of the concept MRFT under the urban driving condition and the suburban driving condition are shown in Figure 7. It can be seen that EV driving in urban or suburban areas for a long time has obvious differences in EV efficiency and the MRFT speed ratio. As far as EV efficiency is concerned, EVs running in urban areas are in a low velocity or static state for a long time, and their efficiency is generally lower than that of EVs running in suburban areas. As for the MRFT speed ratio, EVs driving in urban areas have higher requirements on the MRFT, where (a) the maximum speed ratio is 4.9 and (b) the speed ratio fluctuates around 0.9 in most cases, and 1.7 and 2.1 in a few cases. However, the maximum speed ratio of EVs driving in suburban areas only reaches 2.8, and the speed ratio fluctuates around 0.85 in most cases. This shows that the MRFT of EVs driving in urban areas should preferably have 3 fixed speed ratios, i.e., 0.9, 1.7, and 2.1, and the range of variable speed ratios is 0.8–1, 1.6–1.8, and 2–2.2. In contrast, the MRFT of EVs driving in suburban areas only needs 1 fixed speed ratio of 0.85, and the range of variable speed ratio is 0.6–1.

### 4.2. Feasibility Analysis of the Proposed Strategy

As the battery is both the energy supply and storage device of an EV, the running state of the battery under different SOC and environmental temperatures directly affects the EV’s driving performance. Therefore, the performance of the proposed strategy under different SOCs and environmental temperatures can reveal the strategy feasibility of EVs equipped with MRFT under real driving conditions, i.e., the urban driving condition and the suburban driving condition.

**Figure 8:**
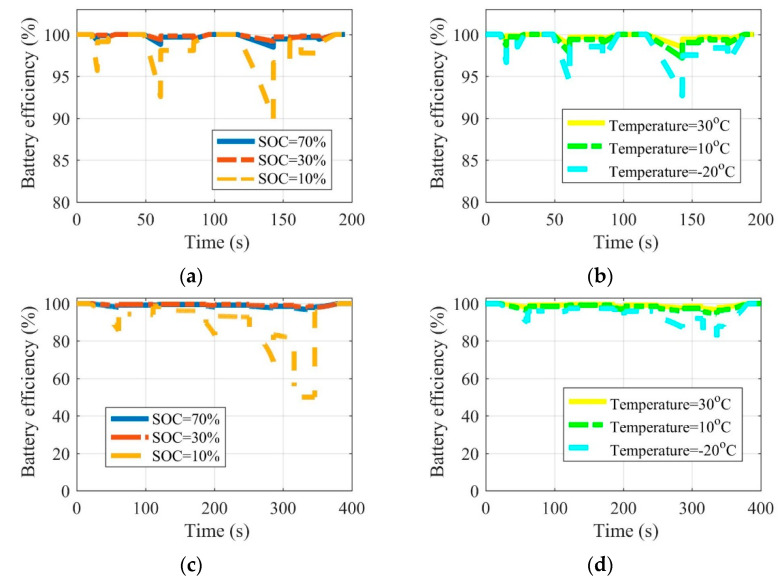
Battery efficiency at different SOCs and environmental temperatures under the urban driving condition (**a**,**b**) and the suburban driving condition (**c**,**d**).

**Figure 9:**
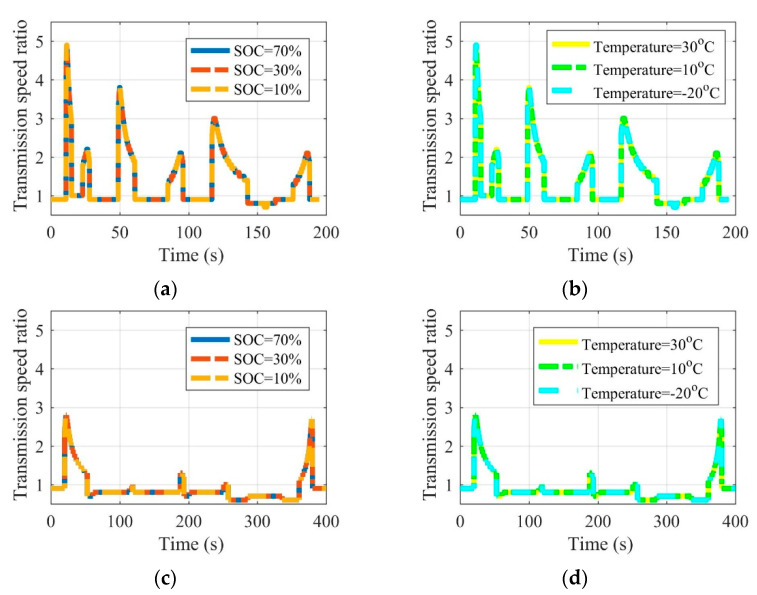
Speed ratio of MRFT at different SOCs and environmental temperatures under the urban driving condition (**a**,**b**) and the suburban driving condition (**c**,**d**).

**Figure 10:**
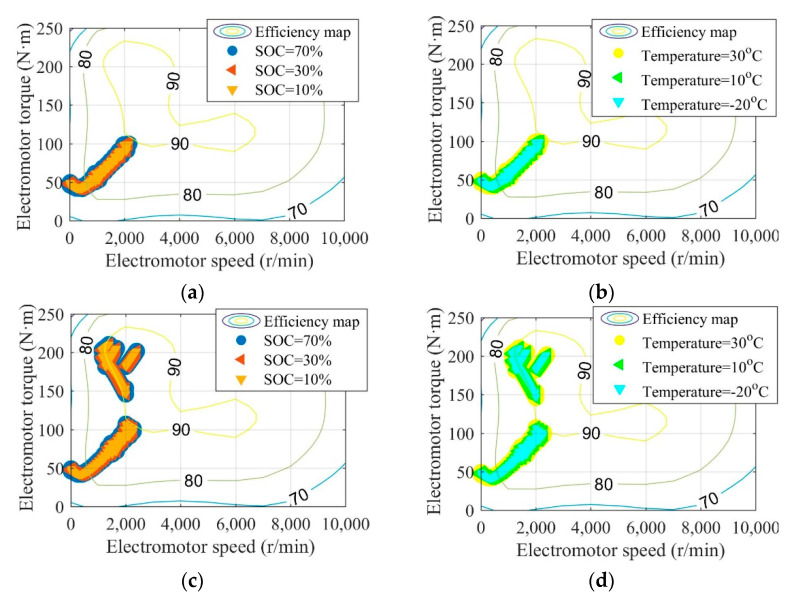
Electromotor operation state under different SOCs and environmental temperatures under the urban driving condition (**a**,**b**) and the suburban driving condition (**c**,**d**).

Figure 8, Figure 9 and Figure 10 are the battery efficiency, the speed ratio of the MRFT, and the electromotor operation state at different SOCs and environmental temperatures under the urban driving condition and the suburban driving condition, respectively. It can be seen that under most SOCs, i.e., 30–70%, and environmental temperatures, i.e., 10–30 °C, EVs equipped with MRFT can maintain relatively stable performance, except under extremely low SOC reaching 10% and environmental temperatures reaching −20 °C. Notably, when the SOC is as low as 10% or the environmental temperature is as low as −20 °C, the only change is the operating state of the battery, and this change does not affect the operating state of other powertrain devices. This is because the powertrain achieves the optimal operation state through the adjustment of the MRFT, and the battery performance does not have a large disturbance to this state with the change of SOC and environmental temperature.

### 4.3. Application Potential Analysis of the Proposed Strategy

The application potential of EVs equipped with MRFT under real driving conditions can be obtained by comparing it with EVs without transmission. The SOC and the environmental temperature are set at 70% and 30 °C, respectively.

**Figure 11:**
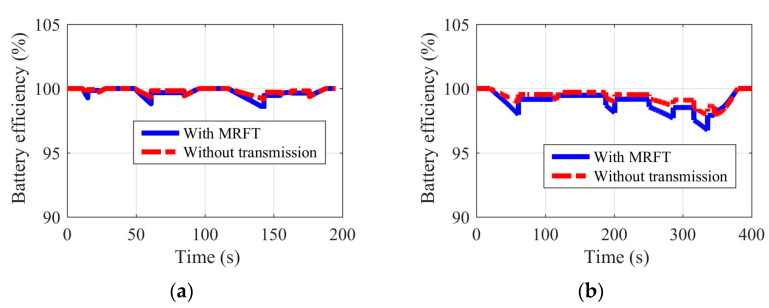
Battery efficiency of EVs equipped with MRFT and without transmission under the urban driving condition (**a**) and the suburban driving condition (**b**).

**Figure 12:**
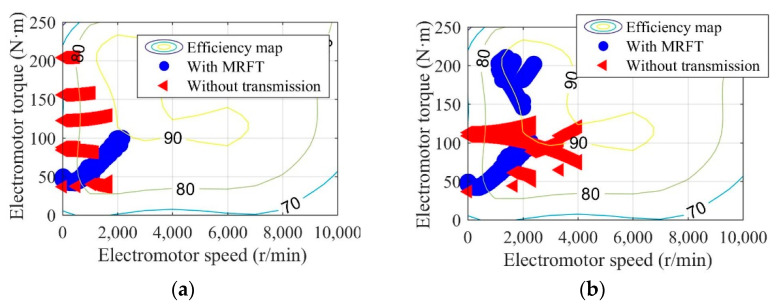
Electromotor operation states of EVs equipped with MRFT and without transmission under the urban driving condition (**a**) and the suburban driving condition (**b**).

**Figure 13:**
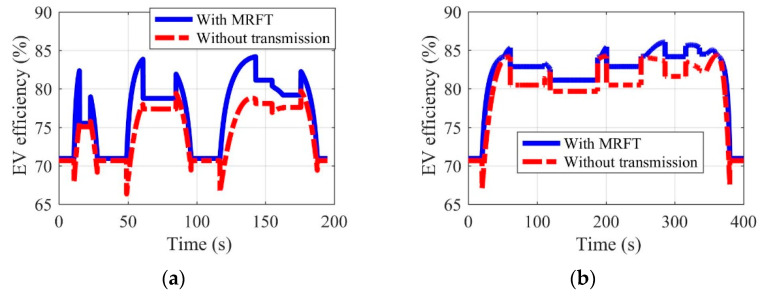
Efficiency of EVs equipped with MRFT and without transmission under the urban driving condition (**a**) and the suburban driving condition (**b**).

**Figure 14:**
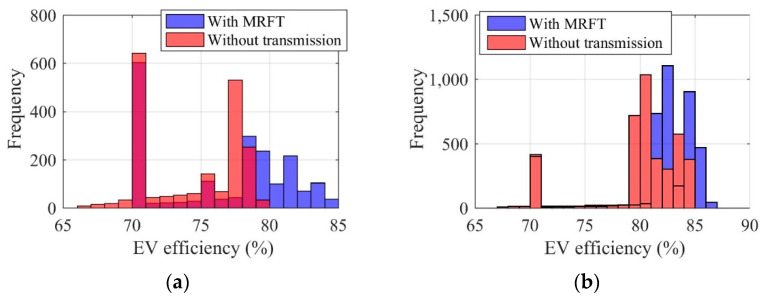
Efficiency statistics of EVs equipped with MRFT and without transmission under the urban driving condition (**a**) and the suburban driving condition (**b**).

Shown in Figure 11, Figure 12, Figure 13 and Figure 14 are the battery efficiency, the electromotor operation states, the EV efficiency, and the efficiency statistics of EVs equipped with MRFT and without transmission under the urban driving condition and the suburban driving condition, respectively. As far as the battery efficiency is concerned, after the MRFT is equipped, battery efficiency decreases slightly. This is because the MRFT has a strong regulatory effect on the whole powertrain, thus it coordinates the operation status of each powertrain device in urban and suburban driving conditions. However, this coordinated control sacrifices battery efficiency. This phenomenon is explained in Figure 12, as the electromotor is a device for energy conversion, and its efficiency is greatly sensitive to speed and torque. After the MRFT is equipped, the operating state of the electromotor is significantly improved, and the operating condition points change from decentralized distribution to centralized distribution. The distribution change of operating condition points greatly improves the EV performance, as shown in Figure 13. Under urban driving conditions, even if the EV without transmission adopts the proposed optimization strategy, compared with that, the efficiency of the EV with MRFT is improved by 9.7% at most and 7.4% under suburban driving conditions. More detailed results can be obtained from Figure 14. Regardless of the static state of the EV, as shown in Figure 14, the efficiency of the EV equipped with MRFT has shifted to the right as a whole in urban and suburban driving conditions, which means the efficiency of most driving time in the urban driving condition has changed from 75–79% to 75–85% and from 79–85% to 81–87% in the suburban driving condition. Notably, for the urban driving condition, the proportion of driving time with efficiency greater than 80% increased from 10.1% to 58.7%, and for the suburban driving condition, the proportion increased from 66.8% to 88.8%.

## 5. Conclusions

With the continuous progress of vehicle electrification, EVs are gradually serving as the green mean of transportation for daily trips. Consequently, EV driving conditions have become increasingly stable and regular. In this context, the driving strategy of EVs that can adapt to driving conditions needs to be put forward urgently. Hence, taking the MRFT with characteristics of both MST and CVT as a breakthrough, this paper proposes an optimization strategy dedicated to driving adaptation for EVs.

This paper starts with the EV dynamics analysis in driving conditions, the powertrain model considering the energy transmission process, and the driving-adapt transmission model considering MRF from the perspective of model and analysis. Then, according to the constructed powertrain model, the driving-adapt optimization strategy in specific driving conditions is proposed. Finally, the simulation results show that (a) compared with the suburban driving condition, the urban driving condition has higher requirements for the MRFT, and the MRFT used in the urban driving condition requires two more fixed speed ratios and variable speed ratio ranges; (b) EVs equipped with MRFT achieve the expected driving performance at most SOCs and environmental temperatures, but when the SOC is as low as 10%, the battery efficiency will fluctuate between 50.0% and 99.2% with the change of EV velocity; and (c) in terms of EVs equipped with MRFT, the operating point of the electromotor will change from decentralized to centralized, and the driving time with efficiency greater than 80% will increase from 10.1% to 58.7% in the urban driving condition and from 66.8% to 88.8% in the suburban driving condition. Thus, the proposed driving-adapt strategy for EVs equipped with MRFT takes both the application and the design of EVs into consideration and provides a solid theoretical basis for mitigating or eliminating many traffic problems caused by the EV powertrain operating characteristics in the vehicle electrification process.

As this paper aims to explore the driving-adapt potential of MRFT on EVs and the critical problem is the combination of the powertrain and driving conditions, the proposed MRFT model qualitatively describes the change of MRFT efficiency in specific conditions. Therefore, in the future, more detailed numerical and theoretical models for the MRFT will be studied.

## Figures and Tables

**Table 1:** The relevant simulation parameters.

Parameter	Value	Unit
M	1600	kg
δ	1.2	
$C_{A}$	0.43	kg/m
g	9.8	${m/s}^{2}$
f	0.028	
R	0.307	m
μ	0.7	
$i_{o}$	3.863	
$v_{\max}$	45	m/s
$v_{\min}$	0	m/s
$a_{\max}$	3	${m/s}^{2}$
$a_{\min}$	−2	${m/s}^{2}$
$c_{\mathrm{battery}}$	1062.2	${\mathrm{Jkg}}^{-1}K^{-1}$
$\eta_{o}$	0.95	
θ	0	

## Data Availability

The data presented in this study are available on request from the authors.
